# Modelling the fear of crime

**DOI:** 10.1098/rspa.2017.0156

**Published:** 2017-07-12

**Authors:** Rafael Prieto Curiel, Steven Bishop

**Affiliations:** Department of Mathematics, University College London, Gower Street, London WC1E 6BT, UK

**Keywords:** perception of security, fear, crime, opinion dynamics

## Abstract

How secure people feel in a particular region is obviously linked to the actual crime suffered in that region but the exact relationship between crime and its fear is quite subtle. Two regions may have the same crime rate but their local perception of security may differ. Equally, two places may have the same perception of security even though one may have a significantly lower crime rate. Furthermore, a negative perception might persist for many years, even when crime rates drop. Here, we develop a model for the dynamics of the perception of security of a region based on the distribution of crime suffered by the population using concepts similar to those used for opinion dynamics. Simulations under a variety of conditions illustrate different scenarios and help us determine the impact of suffering more, or less, crime. The inhomogeneous concentration of crime together with a memory loss process is incorporated into the model for the perception of security, and results explain why people are often more fearful than actually victimized; why a region is perceived as being insecure despite a low crime rate; and why a decrease in the crime rate might not significantly improve the perception of security.

## 1. Introduction

Fear of crime is a feeling, perhaps even a natural instinct, which is based on our perceived risk of suffering a crime. This fear encourages us to avoid certain streets, makes us walk faster during the night and motivates each of us to spend more than one month of our lives locking and unlocking cars and buildings [1] or taking other security measures as part of our daily routine. The reasons why we fear crime in one region more than another may be based on our past experiences in that region, perceived street disorders and incivilities [2], demographic factors of the region [3] and because of the people within the local community [4], among many other factors, but is this fear merely in direct relation to the actual crime? While past victimization increases the probability that a person actually fears crime [5], being the victim of a crime does not completely explain the generalized fear of crime [6] and regions are frequently perceived as being insecure even when they suffer a relatively low crime rate [7]. It is the fear of crime, not necessarily crime itself, that motivates us to take healthy precautions, such as locking the doors of our home [8], and creates awareness and caution [9], but, in extreme cases, fear has a severe impact on the quality of life and it might cause paranoia, tension and social isolation [8], and fear is the reason why millions of people have been displaced from their own home [10,11].

Many studies have tried to understand and quantify the factors that affect a person’s fear of crime [12]. It has been argued that the perception of insecurity emerges as a social problem [13] and it is now becoming one of the main concerns for residents and administrators in almost every large city [14].

Quantifying the perception of security, or the fear of crime, poses a serious challenge. Firstly, the concept itself. Fear of crime has acquired many divergent meanings and it is almost never defined, but it is implicitly considered as the perception that a person is likely to be the victim of a crime [15]. Secondly, the fact that two people consider a particular place to be *insecure* does not mean that they fear the same things or to the same magnitude. However, by imposing a metric for the perception of security and quantifying the fear of crime, we see that many factors affect it, such as demographic factors (e.g. age, race and gender [14,16]), physical factors (the location of a person or a house or vandalism in particular streets, etc. [17,18]), past victimization and more.

Also, although the media play a key role in how a person updates their beliefs and perceptions, there is only a weak relationship between the feelings of insecurity and the media [19]. There are several reasons for this: the majority of the crimes are not actually reported in the media [20], the crimes which are reported tend to have an emphasis on violence or indecency [21,22], the audience selects the media contents they consume and so people who read crime stories are often more prepared for such news [23] and the impact of a specific report depends on whether or not justice is restored [24] and the individual interpretation, often referred to as the ‘reception’ of media [25], and, therefore, it is not clear whether people who read more newspapers or listen more frequently to the radio, for instance, tend to have a lower or higher fear of crime.

Crime has certain properties which make it hard to analyse. Firstly, crime is a rare event and therefore the majority of the population does not actually suffer any crime. Moreover, many of the crimes end up being an attempt (which may still convey the same fear) [6], and for some victims, the impact of suffering a crime might decay rapidly over a few weeks or months and might also be of limited consequences [9]. Finally, crime is highly concentrated in certain places and in certain population groups with some victims tending to suffer more than one crime (repeat victims) [26,27]. Thus, there is a mismatch between crime and its fear [9] and, even when crime rates have dropped considerably in many countries in recent years [28,29], the fear of crime has not experienced the same drop.

Most studies about the fear of crime and the perception of security are static observations of the current situation in a particular region [8], country [3] or group of countries, by analysing the results of victimization surveys, based on different types of questions about the fear of the individual [30]. Elsewhere, detecting those individuals who actually suffered a crime has allowed the impact of direct victimization to be measured [9]. However, little is known about how the collective perception of insecurity emerges, how it changes over time, what is the impact of a crime on the perception of the victims and most importantly, what is the impact of crime on the perception of security of the many non-victims.

From the point of view of the perception itself, one approach is to consider it as an opinion. A variety of conceptual models already exist which help us analyse potential opinion dynamics: how the interactions between people lead to the emergence of a global consensus [31], what is the opinion volatility [32], what is the role of the social network on the dynamics of opinions [33], what is the impact of extreme opinions [34] or how does the opinion of a leadership affect its dynamics [35]. Mathematically, a wide variety of models have been used in the analysis of opinion dynamics, which go from techniques used in epidemiology [36], kinetic models to determine distribution of opinions over time [37], models based on mean field theory (where the impact of all the individuals is simplified into a single averaged effect) [32] and simulating agents [34,38]. These models have been applied in a variety of settings, such as the behaviour of voters [33], the implementation of a specific tool by a scientific community [36], political segregation in the USA [35] and for modelling the spread of misinformation and fake news on the Internet [39].

The main interest generated by opinion models is how an idea is shared among individuals, how they reach a consensus or, under certain circumstances, how polarization or fragmentation of opinions is observed and how does the persuasiveness, assertiveness and supportiveness of different individuals change the dynamics. These models placed the emphasis on the interaction between individuals and the spread of their ideas, but external factors, which might affect their opinion strongly, are usually ignored or modelled as random noise, sometimes referred to as a process of ‘self-thinking’.

Connecting these two fields, the analysis of the fear of crime with the study of opinion dynamics is not straightforward, since crime cannot be ignored or modelled as random noise. Thus, it is vital to understand the external factors which affect the perception of security and hence determine its dynamics.

Here, we propose a model to quantify the dynamics of the perception of security and then simulate the dynamics under a variety of scenarios to mimic different circumstances that are observed in terms of crime and its fear.

## 2. Perception of security model

Let us suppose that the perception of security of *k* of a fixed region (such as a city or a county) is given by a number *s*_*k*_, between 0 and 1, where 1 means the perception is that the region is the most insecure and 0 is when the place is the most secure. We use a continuous approach for the perception of security to quantify different levels in which a person might fear crime, and a simple way to interpret *s*_*k*_ is that it represents the probability that the person considers the region to be insecure and (1−*s*_*k*_) is the probability that considers it to be secure, so a larger value of *s*_*k*_ means that it is more likely that he or she considers that particular region to be insecure. This means that if *s*_*k*_>*s*_*j*_, then the person *k* considers the same place to be more insecure than the person *j*.

A common practice when surveying individuals about their perception of security is to consider binary answers, so individuals can either chose between the answers of ‘secure’ or ‘insecure’ (as in the Mexican Victimisation Survey [40]) or to provide a fear scale (as in the Crime Survey for England and Wales [41]). The perception of security of the whole population *k*=1,2,…,*n* or any subgroup might be summarized by the mean perception of security, *S*, which is also the expected value of the Poisson Binomial distribution when asking each individual a binary question about their fear or considering a specific threshold for the fear scale.

The perception of security of a person may change over time and to represent this, we write $s_{k}^{\left(t\right)}$ and $s_{k}^{\left(t+1\right)}$ for the perception of security at two consecutive time intervals, for instance, from one week to the next, and hence we consider discrete units of time, defining a discrete dynamical system [42,43]. Usually, the length of this step will be given naturally by data. In crime studies, a commonly used time step is weekly periods, although corroboration with data may only be available at yearly intervals.

Here, we consider three reasons why the perception of security of a person might change from one time step to the next: memory loss, suffering a crime and due to the opinion of others. We assume that the three reasons (memory loss, suffering a crime and exchanging opinion with others) are observed in discrete units of time which represent intervals of one week. Thus, the perception of security of *k* is updated according to the general equation

2.1 $$s_{k}^{\left(t+1\right)}=f(s_{k}^{\left(t\right)},\psi_{k}^{\left(t\right)},I_{k}^{\left(t\right)},{\underline{s}}^{\left(t\right)}),$$

where *f* is a function which represents the dependence of the updated perception of security of *k* at the next time step on its current perception of security $s_{k}^{\left(t\right)}$, its memory $\psi_{k}^{\left(t\right)}$, whether he or she suffered a crime in the corresponding time interval (*t*,*t*+1), expressed as $I_{k}^{\left(t\right)}$ and the impact of the perception of others at that time, $\left\{s_{1}^{\left(t\right)},\ldots ,s_{n}^{\left(t\right)}\right\}$, written as a vector ${\underline{s}}^{\left(t\right)}$.

**Figure 1.**
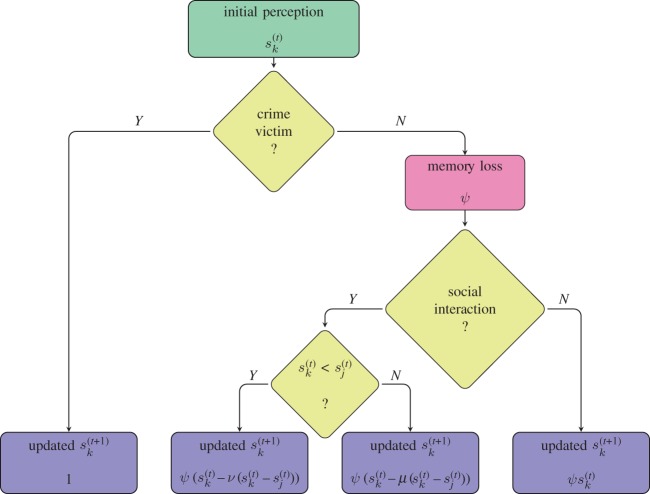
Schematic model in which the perception of security of the person *k* is updated from one week to the next one. There are four possible outcomes depending on whether the person suffered any crime, depending also if the person had an interaction and if that interaction occurred with a more or less fearful person.

We analyse different scenarios which alter the perception of security of *k*, and four different outcomes from each time step are considered. Thus, the role of the function *f* is to update the perception of security of *k* according to the four distinct scenarios, schematically depicted in figure 1. The reasons why different scenarios update the perception of security of *k* differently are explained below.

### (a) Suffering a crime

If the person *k* suffers a crime between *t* and *t*+1, then we consider that the person will naturally think that the place is insecure. More formally, let $X_{k}^{\left(t\right)}$ be the number of crimes suffered by the person *k* between *t* and *t*+1. We take into account the possibility, albeit small, that a person might suffer more than one crime during one time step, so $X_{k}^{\left(t\right)}$ could potentially have large values; therefore, we use a binary variable $I_{k}^{\left(t\right)}=1$ if the person suffered at least one crime between times *t* and *t*+1, and 0 otherwise. Then if $X_{k}^{\left(t\right)}>0$, we assume that $s_{k}^{\left(t+1\right)}=1$ regardless of any previous perception. We then write the impact of crime as

2.2 $$\text{Impact of crime:}\;{}_{\mathrm{crime}}s_{k}^{\left(t+1\right)}=I_{k}^{\left(t\right)}+(1-I_{k}^{\left(t\right)})s_{k}^{\left(t\right)},$$

which is represented as the first step after the initial perception of security in figure 1.

The risk of suffering a crime is not uniformly distributed across the population [44,45] and since our objective is to understand the dynamics of the perception of security and how crime affects it, we need to also consider the distribution of crime across the population. Because of the social environment, activities, age, gender and other reasons, crime is more concentrated in some sectors of the population [46] and in some specific regions [47], giving rise to criminal hotspots [48]. Some groups of people may be ‘immune’ to crime and others may suffer chronic victimization [46]. We consider a distribution of crime which takes into account this inhomogeneous behaviour [49].

Assuming that the number of crimes suffered by a person is independent of others and that suffering a crime does not affect the probability of suffering subsequent crimes, then the number of crimes suffered by the *k*th person $X_{k}^{\left(t\right)}$ might be modelled as a Poisson distribution with rate λ_*k*_≥0. These two assumptions, the independence between the crime suffered by individuals and a constant rate of suffering a crime, may be unrealistic but it enables us to characterize the victimization profile of the whole population (i.e. to consider the λ_*k*_ for each individual), to take into account an immune group (with λ_*k*_=0) and a chronically victimized (with λ_*k*_ large) one and to control the expected number of crimes suffered by the whole population ($\sum_{k}\lambda_{k}$). The victimization profile [50] allows us to simulate the number of crimes that individuals might suffer, so we can compare the perception of security among people who suffer higher or lower amounts of crime.

More complex models for the distribution of the number of crimes suffered by an individual could be considered, but, for now, a simple Poisson model with a constant rate is adopted.

### (b) Memory of past perception

If the person *k* does not suffer a crime between *t* and *t*+1 and everything else remains fixed, then we assume that the person will consider that security is improving, that is the memory of past perception is gradually lost. Thus, by isolating the effect of memory from any other factor, we consider that

2.3 $$\text{Impact of memory:}\;{}_{\mathrm{memory}}s_{k}^{\left(t+1\right)}=\psi_{k}s_{k}^{\left(t\right)},$$

with *ψ*_*k*_∈(0,1), which represents the (constant in time) speed at which the person *k* has a loss of memory, where *ψ*_*k*_ closer to 1 means that the perception of security remains the same at the next step, and a value of *ψ*_*k*_ closer to 0 means that the person forgets their past impressions quickly. This expression is referred to as having *exponential decay* and so, with no other factors, the perception of security will always decay as time goes by.

Here, we assume that all individuals have the same memory loss, and that memory loss is the same for all types of crime, with rate *ψ*, although different speeds at which individuals tend to forget their past perception, based perhaps on the type of crime, could be considered. The effect of memory loss is considered for the non-victims in figure 1.

### (c) Opinion dynamics

If the person *k* interacts with a ‘fearful’ individual, then it is likely that the perception of *k* will be changed by the interaction. Fear is contagious and the impact of an interaction might depend on the closeness between the individuals or the strength of their ideas (or its intensity) [51] both concepts being assessed appropriately. This situation is usually modelled as two people who have different opinions who reach a state closer to each other [52], or closer to a *consensus* opinion after they update their beliefs [37].

In opinion dynamics, a certain amount of random pairs of individuals might be deemed to have an interaction between them [34] or all individuals might update their beliefs simultaneously [38]. In terms of the fear of crime, we consider that not all individuals have an interaction with others each week and so, here, only a certain proportion of the population, *γ*, forms pairs of individuals without replacement, and they share and update their opinions.

It is worth considering that the perception of security differs from other types of opinions, like a left or right political leaning, since we assume that the impact is not symmetric: a more fearful person might share their own experiences with others, increasing the fear of crime in them, without it reducing his or her own fear. Thus, there is an opinion-dependent asymmetry [38], which might be modelled as follows. Let $s_{k}^{\left(t\right)}>s_{j}^{\left(t\right)}$; so the person *k* considers the region to be more insecure than the person *j*. Then, isolating this effect:

2.4 $$\text{Impact of social dynamics on }k:\;{}_{\mathrm{interaction}}s_{k}^{\left(t+1\right)}=s_{k}^{\left(t\right)}-\mu (s_{k}^{\left(t\right)}-s_{j}^{\left(t\right)})$$

and

2.5 $$\text{Impact of social dynamics on }j:\;{}_{\mathrm{interaction}}s_{j}^{\left(t+1\right)}=s_{j}^{\left(t\right)}-\nu (s_{j}^{\left(t\right)}-s_{k}^{\left(t\right)}),$$

where *μ*∈(0,1) is a parameter which might be considered to be the *resistance* of the perception of insecurity and *ν*∈(0,1) is the parameter for the *impact* of the perception of insecurity. Thus, we consider that *μ* is close to zero and *ν* is close to one, so that the person who fears crime the most retains nearly the same perception at the next time step and this fear has a large impact on the other person. Having or not having a social interaction and sharing perceptions with a person who has more or less fear is the last part of figure 1.

A simplification to the model could be achieved by assuming that the resistance of the perception of insecurity is negligible (with *μ*=0) or by considering the relative influence of the two parties so that *ν* and *μ* could be combined into a single parameter, but this simplification has its drawbacks since the parameter *μ*, although small, allows us to take into account the impact of any social support given to the victims of crime and to the persons that fear crime the most [53]. Furthermore, to also mirror the recent models of opinion dynamics [35], we retain both parameters in our model.

With *ν*>*μ*, there is no conservation of the total perception of insecurity [37], that is $s_{k}^{\left(t+1\right)}+s_{j}^{\left(t+1\right)}\geq s_{k}^{\left(t\right)}+s_{j}^{\left(t\right)}$ and there is a certain degree of compromise between individuals, so that $|s_{k}^{\left(t+1\right)}-s_{j}^{\left(t+1\right)}|\leq |s_{k}^{\left(t\right)}-s_{j}^{\left(t\right)}|$. In this way, fear of crime is considered to be a contagious process [54].

After defining the microscopic level of interactions between individuals, a technique used to model the dynamics of the whole population is to consider the distribution of opinions at a certain time *P*(*s*,*t*) and, by applying methods of kinetic theory of binary interactions, to obtain a Boltzmann-type equation [37], or to consider a typical individual and analyse their perception using mean field theory [33]. However, such a system is difficult to study, particularly with factors such as crime or memory, which do not depend on the social dynamics. A commonly used technique for this type of problem is to consider simulated agents [38], located perhaps on a lattice [34] or in a network, where the individuals are represented by the nodes and the edges are the potential interactions between them. In the particular case of opinion dynamics, it is common to consider the effect of a small-world network [55] or a scale-free phenomena [56]; however, our main interest is not the impact of the topology of the social network and therefore we just consider random pairs of individuals.

In the modelling process, individuals alter their perception of security based on their memory loss (equation (2.3)); the perception of others (equations (2.4) and (2.5)); and whether individuals might or might not suffer crime (equation (2.2)). This procedure is then repeated at each time step, updating the model in that specific order so that the social interactions occur after a period of memory loss and crime occurs after the social interactions which completely defines the model for the dynamics of the perception of security. The four possible outcomes from each time step are schematically depicted in figure 1.

There are other factors which might play a significant role in the perception of security, for instance, a particular crime that is well reported in the media. This effect could be easily integrated into the model by adding low-frequency shocks which increase the global fear or that of a selected group, such as the readers. For now, we ignore the impact of the media, and we only consider the impact of memory loss, suffering crime and opinion dynamics in the model of the fear of crime.

## 3. Numerical simulations

### (a) Simulating crime in a population

**Figure 2.**
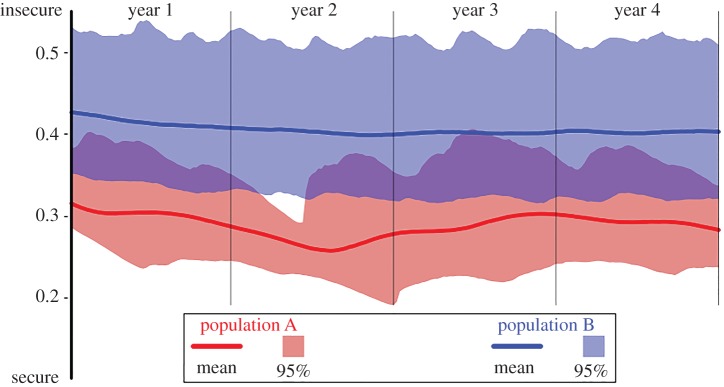
Simulated perception of security of two populations who suffer exactly one crime per week among their 10 000 individuals, with *μ*=0.1, *ν*=0.9, *ψ*=0.5. Population A is a special case where a fixed individual suffers all the (weekly) crimes and in population B crime is randomly suffered. On average, population B has a mean perception of security 0.12 above population A, even when they both suffer the same amount of crime and have the same dynamics.

Without crime and memory, individuals who share their opinions eventually reach a consensus [34], meaning that all the opinions end up being close to one another. Therefore, what is relevant in this model is the impact of these two elements, crime and memory. Crime is not suffered randomly by the population and therefore it should not be modelled simply as a homogeneous variable or noise. The distribution of crime rates, λ_*k*_, plays a fundamental part in the model. Comparing, for instance, the mean perception of security of two populations who suffer exactly the same amount of crime but with a different distribution (figure 2) reveals that results are highly dependent on how the distribution of the crime suffered is modelled.

To mimic a more realistic distribution of crime, from here onwards we assume that the victimization profile can be described as a mixture model [50]. The number of crimes suffered by a random person is given by

3.1 $$q_{1}\;\text{Pois}(\lambda_{1})+q_{2}\;\text{Pois}(\lambda_{2})+\cdots +q_{m}\text{Pois}(\lambda_{m}),$$

which means that the individual is allocated into one of the *m* groups (with probability *q*_*j*_) and then the number of crimes that he or she suffers has a Poisson distribution with the corresponding rate λ_*j*_. Crime rates are reported here on a yearly rate due to their extreme low frequency, but the weekly rate can easily be computed.

For the numerical simulations, we use *m*=3 groups, with $\bar{q}=(0.65,0.3,0.05)$ and a yearly rate $\bar{\lambda }=(0,0.05,1.7),$ meaning that we assume that 65% of the population suffers no crime (group 1), with λ_1_=0, then 30% of the population suffers crime at a low (yearly) rate λ_2_=0.05 (group 2) and 5% of the population suffers crime at a higher yearly rate of λ_3_=1.7 (group 3). Under this victimization profile, the population expects to suffer 10 crimes for every 100 people each year, and 65% of the population is immune to crime. A simpler model could be obtained by considering only victims and non-victims (that is, only two groups), but crime is not a simple process and evidence shows that we frequently observe different degrees in which crime is suffered, ranging from people who are statistically immune to crime [46] to people who suffer a small amount of crime and, finally, a small population group which suffers a much higher rate [50] so that a more realistic victimization profile is obtained with more than two groups.

### (b) Simulating the interactions of individuals

We simulate the perception of security of a population with *n*=10 000 individuals who update their perception of security each week, who have memory loss (equation (2.3)), might suffer a crime (equation (2.2)) and might alter their opinion based on the perception of others (equations (2.4) and (2.5)). From the simulations, we report the mean perception *S*^(*t*)^ of each group.

During each step, 10% of the population (1000 individuals) are randomly selected to interact with another 10% of the population, so that during each step 1000 distinct pairs with no replacement are made with the individuals sharing their perspective with each other. Individuals who are not picked to interact with others simply update their perception of security according to the memory and crime rules. The level of interactions, thus, is *γ*=0.2, meaning that 20% of the individuals have an interaction during each time step.

The simulated individuals begin with a random perception of security and we run the algorithm for 6 simulated years and discard the first 2 years to reduce the impact of the initial random perception.

Considering different victimization groups allows us also to measure their degree of mixing, or their *homophily* H [57], defined as the proportion of times that interactions occur between a pair of individuals from the same group. With *m*=3 groups, with respective sizes $\bar{q}=(0.65,0.3,0.05)$, a value of the homophily H=0.631±0.003 occurs when interactions happen randomly; higher values mean that individuals have *preferred* interactions with people from their own group, (so that they interact primarily with people who suffer a similar crime rate) and lower values mean *discouraged* interactions with people from their own group (so interactions occur between people who suffer different crime rates).

## 4. Results

**Figure 3.**
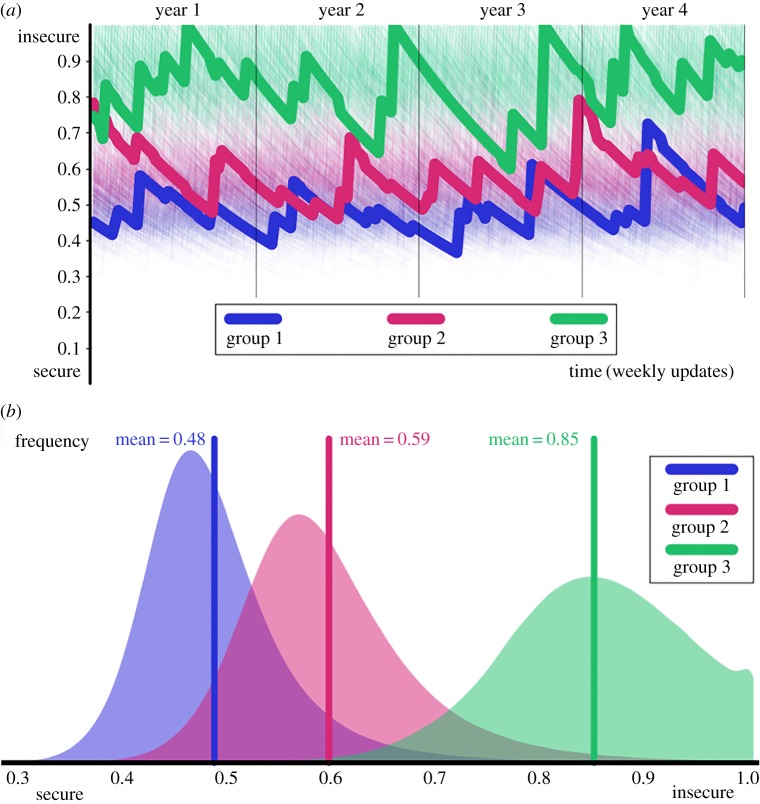
Simulated perception of security with *μ*=0.1, *ν*=0.9, *ψ*=0.5, preferred interactions (homophily H=0.981) and crime rates $\bar{\lambda }=(0,0.05,1.7)$. (*a*) 500 individuals from each group and three representative members highlighted. The highlighted individual from group 3 suffered four crimes (when *s*_*k*_=1); the other jumps are due to the social interactions; the slow decay is the memory loss. (*b*) The distribution of the perception of security for all individuals observed during the 4 years.

The three groups considered have a different distribution of their perception of security (figure 3). Even the group immune to crime (group 1) has a mean perception of security of 0.47, so nearly half of their population fears crime.

The effects of the parameters of the model is, perhaps, as expected: a value of *ψ* closer to 1 means that the population has more memory and, therefore, the perception of insecurity remains for a longer period. A higher value of the impact of insecurity *ν* increases the overall perception of insecurity, and similarly, but with the opposite effect, with the resistance of insecurity *μ*. A higher level of interactions *γ*, when *ν*>*μ*, also increases the mean perception of insecurity.

### (a) Impact of suffering more or less crime

**Figure 4.**
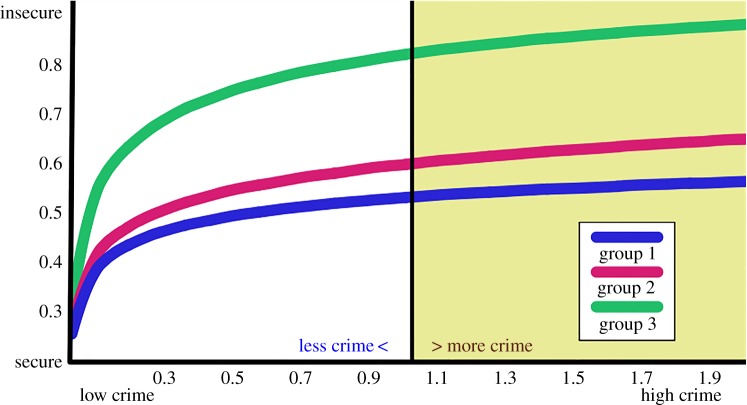
Simulated perception of security with *μ*=0.1, *ν*=0.9, *ψ*=0.5, preferred interactions (homophily H=0.951±0.002) and with yearly crime rates $\kappa \bar{\lambda }=(0,0.05\kappa ,1.7\kappa )$, with *κ* on the horizontal axis. Crime increases with *κ*>1 and decreases with *κ*<1.

There are some surprising results from the model. Firstly, since the amount of crime suffered by the population might change, we measure the impact of these fluctuations by considering a *factor* *κ*>0, and simulate a population which suffers a yearly rate $\kappa \bar{\lambda }=(0,0.05\kappa ,1.7\kappa )$, so that the crime, even when it increases or decreases, maintains the same distribution (figure 4). Thus, with *κ*>1 crime increases and with *κ*<1 it decreases.

A relevant result is that if crime doubles in frequency (with *κ*=2) or, similarly, if crime drops to a half (with $\kappa =\frac{1}{2}$), the mean perception of security of the whole population (from each one of the three groups) undergoes only a slight variation in a seemingly linear manner (figure 4). Approximately, an increase in *κ* of the crime rates increases the perception of insecurity by 0.035(*κ*−1). A drastic (nonlinear) change, though, is observed when *κ*<0.2, which is the threshold after which the group that suffers the highest amount of crime (group 3) actually experiences less crime and shares this with the other groups.

Thus, to improve the perception of security of a population, crime has to decrease considerably. The impact of a slight reduction in crime is negligible.

### (b) Impact of having more or less homophily

Homophily plays a key role in the dynamics of the perception of security. For people who initially are not victims of crime and do not perceive insecurity, as they interact more with individuals from other groups (that is, as homophily is reduced), the perception that the region is insecure increases.

**Figure 5.**
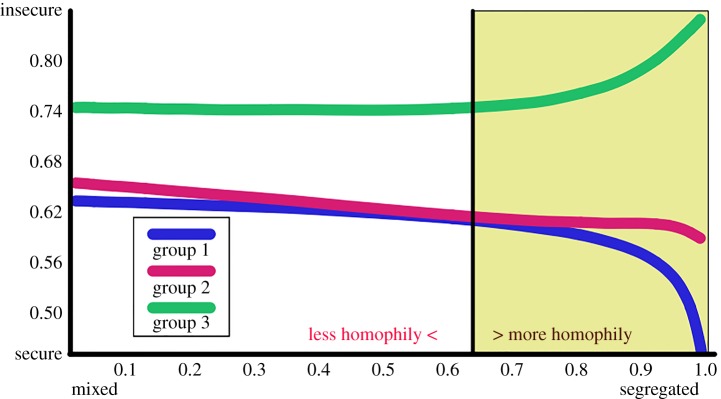
Mean perception of security of a population with *μ*=0.1, *ν*=0.9 and *ψ*=0.5. Pairwise interactions occur between the three groups with a degree of preference or rejection to interactions with their own population group which gives values of homophily H between 0 and 1. Higher homophily means that interactions occur between members of the same group. The vertical line, where homophily H=0.631, represents random interactions. With random interactions, the mean perception of groups 1 and 2 is nearly the same, but it changes considerably on both extremes of the homophily range.

A value of the homophily H=0.631±0.003 occurs when interactions occur randomly, but modelling preferential or discouraged interactions between members of the same group allows us to measure the impact that a more or less homophilic dynamic has on their mean perception of security (figure 5). Under fully mixed population groups (random interactions) there is a fragmentation of the mean perception of security observed among individuals who suffer zero or close to zero crime and individuals who suffer higher crime rates [31,34].

When the homophily is equal to one, individuals only interact with members of their own group, and each group can be analysed separately. The results show that the mean perception for group 3 is *S*_3_=0.858±0.010 and for group 2 is *S*_2_=0.577±0.014, which implies that in a population in which only 5% of their members expects to suffer one crime annually (group 2), the perception of insecurity is already quite high (approx. 60%). Thus, even with the low frequency of crime, we observe a high level in the perception of insecurity. For the members of group 1, who have no reason to perceive insecurity since their members do not suffer any crime and they do not obtain a perception of insecurity from others, their mean perception decreases from their initial values to zero. However, as soon as the members from group 1 interact with individuals of groups 2 and 3, their perception of insecurity rapidly increases. For instance, with an homophily of 0.99 (which means that only 1% of the interactions occur between individuals of different groups) the mean perception of security of group 1 is already *S*_1_=0.412±0.005. Only a few interactions between individuals who belong to a different group is enough to create a fear of crime in 41% of the population of the group which will not, in fact, suffer any crime. Thus, the perception that a region is secure is quite unstable.

## 5. Conclusion

A model for the dynamics of the perception of security (or insecurity) of a region allows us to draw conclusions about the way in which the idea that a region is insecure might be shared in a population. Our focus, rather than being on the dynamics of the ideas (the perception of security) itself, is on the particular attributes of crime and its impact which need to be considered in a model that captures the dynamics of the perception of insecurity. Crime is rare and highly concentrated and, therefore, the majority of the population will rarely suffer any crime but, nonetheless, will have the fear of crime to some degree.

The construction of a model in which agents follow three simple rules in terms of their perception of security (memory loss, sharing of their opinion and the possibility of suffering a crime) roughly mimics the way in which social fears, such as the perceived insecurity, might be spread. The results obtained by this model help us explain why we persistently observe cities and countries in which crime rates are fairly low but yet there is still a generalized fear of crime.

Using different levels of the global rates of crime, the results of this research reveal that these global rates need to decrease considerably to improve the mean perception of security of a region. Small variations of the global crime rates have a negligible impact on the generalized fear of the population.

A metric for the interaction between individuals who suffer different levels of crime, the homophily, has a strong impact on the perception of security, particularly when the interactions between individuals from different groups are scarce. With this new model, we are able to show that only a few interactions between diverse people are enough to create chronically worried population groups, consisting of people who will not actually suffer any crime but nonetheless fear it. Thus, the perception that a region is secure is quite unstable and might change drastically with only a few criminal incidents.

The results presented here are a useful stage in the analysis of the fear of crime and its dynamics, but the complete behaviour is far from being fully explained and further steps are required. Firstly, there is a need to corroborate the model with the observed fear of crime, perhaps obtained through vicitmization surveys, which could provide some insights into benchmark parameters of the model, although verifying the dynamics with actual data poses a challenging task, starting with the choice for the level of homophily and the amount of interactions between individuals. Information technologies might potentially deliver suitable data.

A similar technique could also be used for modelling the dynamics of other types of public opinion which also deserve attention: the fear caused by terrorism or the public opinion of international migration. In both of these cases, the opinions and perceptions of others, the effects of mass media and the impact of misinformed perceptions could be the main reasons why our opinion changes, rather than actual facts.

We show that the fear of crime can be considered to be contagious, that the perception that a region is secure is quite unstable, that a decrease in the crime rates might have almost no effect on the perception of security and that large population groups who are immune to crime might yet be seriously concerned about crime. Thus, a simple model for the dynamics of the fear of crime helps us to gain a better understanding of the frequent mismatch between crime and its fear.

## Data accessibility

This work does not have any experimental data.
